# The Essentials of Bacteriology

**Published:** 1892-09

**Authors:** 


*The Essentials of Bacteriology.* By M. V. BALL, M.D. Pp. xii., 159. Philadelphia: W. B. SAUNDERS. 1891.

This book gives an outline of the structure of bacteria, their methods of growth, &c., and the details necessary for their examination in the laboratory. The most important and well-known varieties are dealt with separately, and sufficient information is given for their identification. A short appendix is added on yeasts and moulds, with an account of the apparatus used in the examination of air, soil, and water.

The author does not claim any originality, but states that his work is somewhat superficial. It is evident, however, that he is practically acquainted with the subject, and the book will, no doubt, be useful. The illustrations are to the point and the coloured plates are well printed.

There are, of course, omissions; in a condensation such as this they are not to be wondered at. The style (with the exception of certain Americanisms, such as "right here," meaning "now," &c.) is clear and intelligible.

